# Vesicovaginal Fistula Repair Using Amniotic Membrane Interposition: A Case Report

**DOI:** 10.7759/cureus.113837

**Published:** 2026-08-03

**Authors:** Achraf Benamou, Anouar El Moudane, Anass El Alaoui, Marouan Chafi, Ali Barki

**Affiliations:** 1 Department of Urology, Faculty of Medicine and Pharmacy of Oujda, Oujda, MAR; 2 Department of Urology, Mohammed VI University Hospital, Oujda, MAR

**Keywords:** amniotic membrane, case report, fistula repair, reconstructive urology, regenerative medicine, vesicovaginal fistula

## Abstract

Vesicovaginal fistula (VVF) is a debilitating condition associated with significant physical, psychological, and social morbidity. Surgical repair remains challenging because recurrence is often related to poor tissue quality and impaired wound healing. Human amniotic membrane (AM) possesses anti-inflammatory, anti-fibrotic, antimicrobial, and regenerative properties that may enhance tissue repair.

We report the case of a 51-year-old woman who developed continuous urinary leakage following total hysterectomy for uterine fibroids. Clinical examination and cystoscopy revealed a 10-mm high vesicovaginal fistula located at the vaginal cuff. The patient underwent transabdominal surgical repair with interposition of an amniotic membrane patch obtained from a local tissue bank after donor screening and informed consent. The fistulous tract was excised, and the bladder and vaginal defects were closed separately using absorbable sutures with AM interposed between the repair layers.

Postoperative recovery was uneventful, and the patient was discharged on postoperative day 4. At the two-week follow-up, she remained asymptomatic with no evidence of urinary leakage or fistula recurrence.

This case suggests that amniotic membrane interposition may represent a safe and feasible adjunct in vesicovaginal fistula repair by promoting tissue healing and reducing recurrence risk. Further studies with larger patient populations and longer follow-up are needed to better define its role in reconstructive urologic surgery.

## Introduction

Vesicovaginal fistulas (VVFs) are rare complications, predominantly of iatrogenic origin. They are associated with significant morbidity and profound impairment in patients’ quality of life [1].

Surgical management of VVFs remains a major challenge for urologists, mainly because of the risk of recurrence following fistula repair. Surgical success largely depends on the quality of the surrounding tissues, which should be supple, non-fibrotic, and well vascularized [2].

The biological characteristics of the amniotic membrane (AM), together with its successful use in reconstructive ophthalmologic surgery, have encouraged its application in reconstructive urology with the aim of promoting optimal healing and reducing recurrence rates [3,4].

The aim of this report is to highlight the potential role of amniotic membrane interposition in vesicovaginal fistula repair through our initial experience at the Urology Department of Mohammed VI University Hospital in Oujda.

## Case presentation

A 51-year-old woman presented with continuous urinary leakage through the vagina following a total hysterectomy performed for uterine fibroids in September 2024. Histopathological examination revealed no evidence of malignancy.

Clinical examination and cystoscopy identified a high vesicovaginal fistula measuring approximately 10 mm at the level of the vaginal cuff. A transabdominal surgical repair with interposition of an amniotic membrane patch was planned.

**Figure 1:**
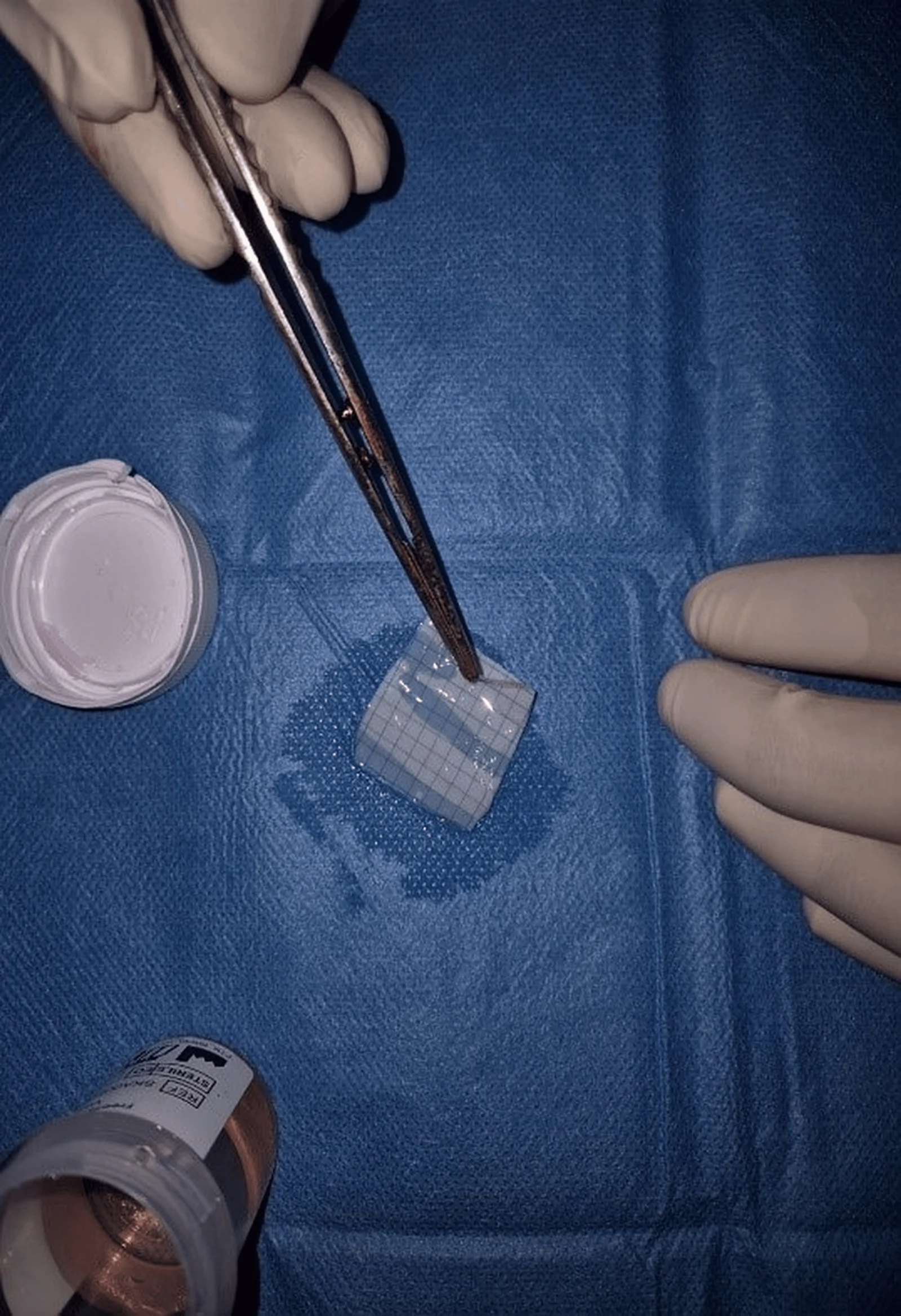
Preparation of the amniotic membrane before insertion between the vesical and vaginal orifices

**Figure 2:**
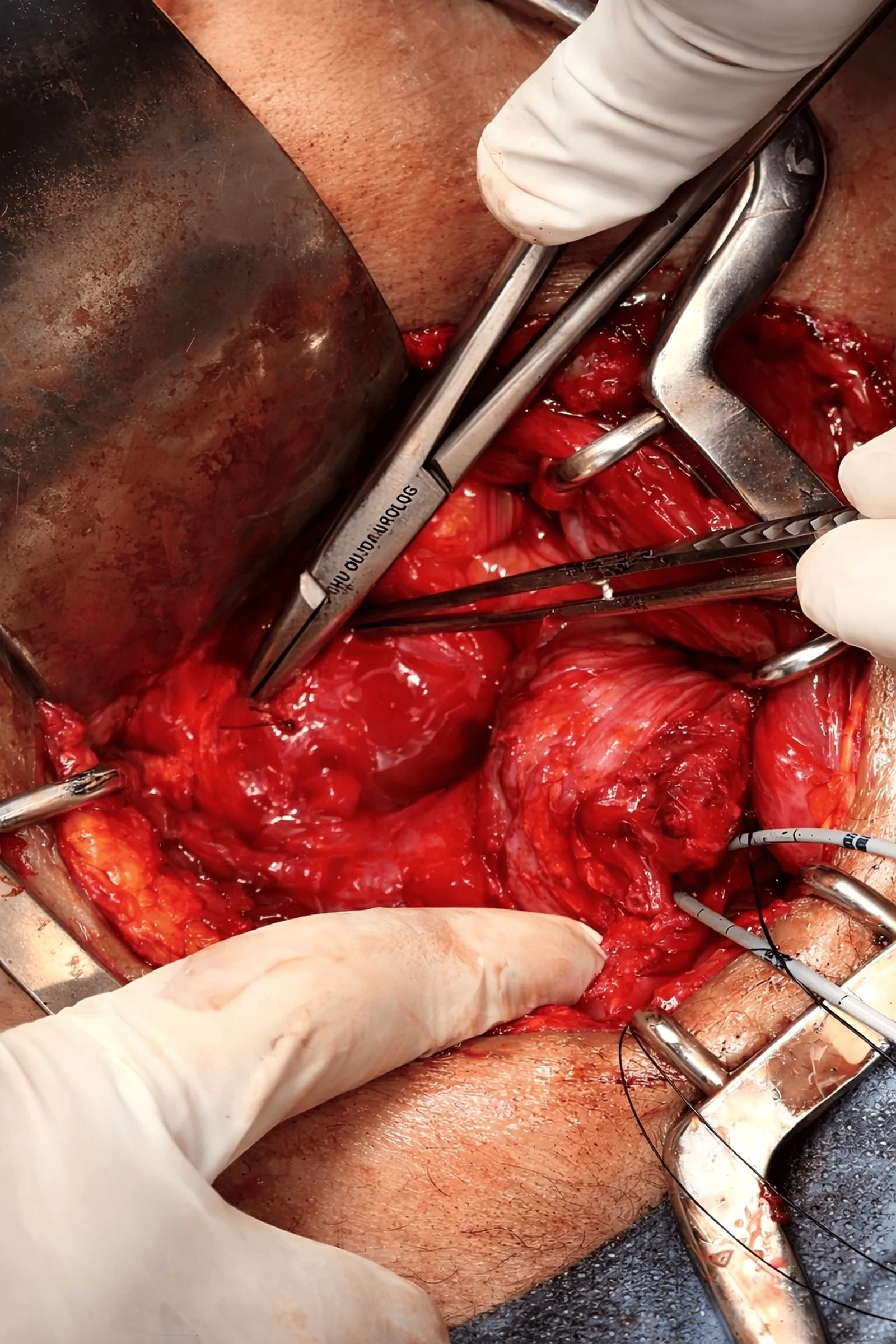
Interposition of the amniotic membrane between the vaginal and bladder fistula openings

The amniotic membrane was obtained from a local tissue bank following informed donor consent and complete serological screening. Surgery was performed under general anesthesia through a median infraumbilical laparotomy. After cystotomy, the fistulous tract was identified, and the fibrotic tissue was excised. The vaginal and bladder defects were then closed separately using absorbable sutures. An amniotic membrane patch was interposed between the two suture lines. A Foley catheter was left in place for 10 days (Figure 1 and Figure 2).

**Figure 3:**
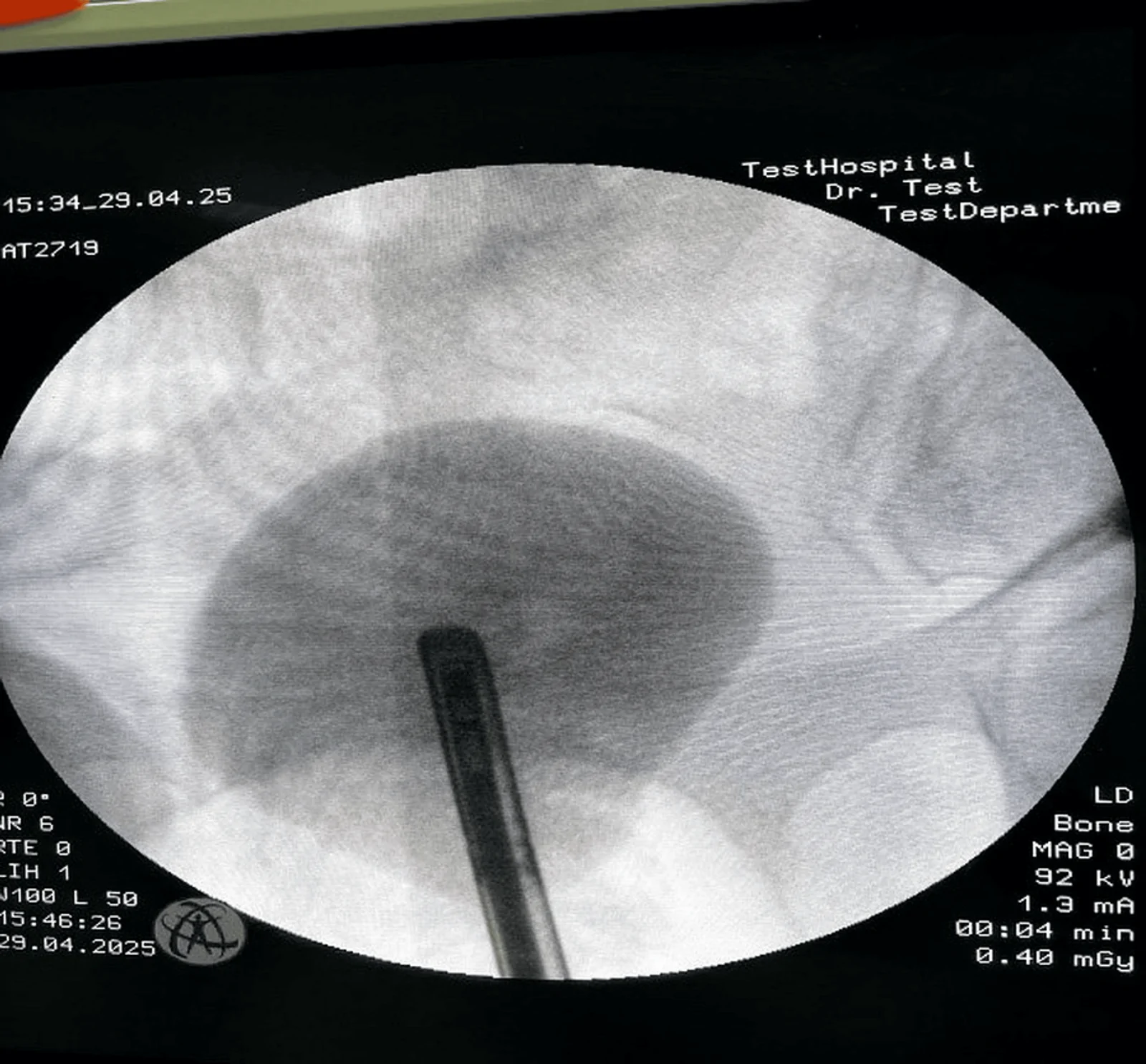
No extravasation of contrast medium during the control cystography

Postoperative recovery was uneventful. The patient was discharged on postoperative day 4. At the two-week follow-up visit, she was asymptomatic with no urinary leakage, and clinical examination confirmed complete healing (Figure 3).

## Discussion

The human amniotic membrane was first described by Davis for the treatment of severe skin burns and chronic non-healing wounds [2]. Subsequently, ophthalmologists adopted its use, and since the 1910s, amniotic membrane allografting has become a recognized therapeutic option for corneal ulcers and ocular surface lesions. Its application in reconstructive urology emerged in the 1990s, particularly in urethral surgery, with encouraging results.

The efficacy of amniotic membrane in vesicovaginal fistula repair has also been reported in the literature, notably by Price and Price [3]. In 2015, they described the case of a patient with a VVF secondary to cervical cancer treated with radical hysterectomy followed by radiotherapy and chemotherapy. Repair using amniotic membrane interposition resulted in favorable outcomes without recurrence during follow-up.

The placenta is a temporary organ of fetal origin that plays a critical role during pregnancy by facilitating maternal-fetal exchange and acting as a natural graft tolerated by the maternal immune system. Structurally, it consists of a fetal (chorionic) surface and a maternal (decidual) surface. The amniotic membrane represents the innermost layer of the fetal membranes and is in direct contact with the amniotic fluid [5].

One of the main challenges in reconstructive urology is achieving durable functional outcomes without recurrence. The amniotic membrane possesses multiple biological properties that are essential for optimal wound healing [6]: promotion of epithelialization through enhancement of epithelial cell growth, migration, differentiation, and adhesion mediated by growth factors such as epidermal growth factor (EGF), hepatocyte growth factor (HGF), and keratinocyte growth factor (KGF); anti-fibrotic and anti-inflammatory effects through the inhibition of transforming growth factor-beta (TGF-β), secretion of IL-10, and suppression of pro-inflammatory cytokines; regulation of angiogenesis mediated by factors such as endostatin, tissue inhibitors of metalloproteinases (TIMPs), and thrombospondin-1; antimicrobial activity related to the presence of lysozyme, lactoferrin, and defensins; and immunomodulatory properties associated with low expression of human leukocyte antigen (HLA) class II antigens and absence of co-stimulatory molecules.

The human placenta is readily available, although its collection requires informed consent, most commonly after planned cesarean delivery. Donor screening includes serological testing for HIV (Human immunodeficiency virus), hepatitis B and C, human T-cell lymphotropic virus (HTLV), syphilis, cytomegalovirus (CMV), and tuberculosis [7].

Several methods of amniotic membrane preservation have been described, each potentially affecting its biological properties [8].

Fresh amniotic membrane is harvested and used without preservation, thereby retaining native growth factor concentrations [9,10], as well as excellent elasticity, adhesion, and transparency.

Cryopreservation, introduced by Kim and Tseng in 1995 [11], preserves proteins, extracellular matrix components, and basement membrane structure at very low temperatures (approximately -80°C), allowing storage for up to 1-2 years, and up to 5 years at -140°C. Although cellular viability does not exceed three months, cryopreserved AM maintains a histological structure and thickness similar to fresh tissue, albeit with increased fragility.

Lyophilization involves dehydration by sublimation after rapid freezing (-50°C to -80°C), reducing water content to 5%-10% and allowing storage at room temperature for up to 5 years [12]. Although its mechanical strength is lower than that of fresh or cryopreserved membranes, some growth factors, such as EGF and TGF-β1, may be better preserved [9,10], making it suitable for large tissue defects.

Air-dried amniotic membrane is prepared under laminar flow at room temperature and sterilized by gamma irradiation, allowing easy long-term storage. It demonstrates membrane stability comparable to frozen fresh AM and may contain higher levels of EGF [13,14].

VVFs remain among the most significant complications of gynecologic and obstetric surgery, with an incidence ranging from 0.3% to 3% [10]. In developing countries, VVFs are predominantly of obstetric origin, whereas in developed countries, approximately 90% are iatrogenic, with up to 75% occurring after hysterectomy. Other causes include digestive or urologic pelvic surgery and post-radiation injury, accounting for 1%-5% of cases [4].

Several innovative techniques have been described to reduce fistula recurrence, which is frequently associated with severe impairment in quality of life and social isolation. Although approaches such as platelet-rich plasma injection into the vaginal wall have been explored, experience with amniotic membrane interposition remains limited. Larger case series and prospective studies are required to confirm the effectiveness and reproducibility of this technique.

The present report has certain limitations, including the description of a single clinical case and the relatively short postoperative follow-up. Nevertheless, the favorable early outcome observed suggests that the amniotic membrane may represent a valuable adjunct in reconstructive urologic surgery.

## Conclusions

In conclusion, amniotic membrane interposition appears to be a safe, feasible, and promising adjunct in the surgical repair of vesicovaginal fistula. Thanks to its anti-inflammatory, anti-fibrotic, antimicrobial, and regenerative properties, it may enhance tissue healing, improve vascularization, and provide an additional protective barrier between repaired tissues, thereby potentially reducing postoperative complications and fistula recurrence, particularly in complex or recurrent cases with poor tissue quality. The encouraging outcome observed in this case suggests that the amniotic membrane could represent an innovative and valuable alternative or complementary interposition material in reconstructive urologic surgery. However, despite these promising preliminary results, larger prospective studies with longer follow-up are still needed to confirm its efficacy, safety, reproducibility, and overall role in contemporary urologic reconstructive practice.

## References

[1] Margolis T, Mercer LJ (1994). Vesicovaginal fistula. Obstet Gynecol Surv.

[2] Davis JS (1909). II. Skin grafting at the Johns Hopkins Hospital. Ann Surg.

[3] Price DT, Price TC (2016). Robotic repair of a vesicovaginal fistula in an irradiated field using a dehydrated amniotic allograft as an interposition patch. J Robot Surg.

[4] Kruse FE, Joussen AM, Rohrschneider K, You L, Sinn B, Baumann J, Völcker HE (2000). Cryopreserved human amniotic membrane for ocular surface reconstruction. Graefes Arch Clin Exp Ophthalmol.

[5] Niknejad H, Peirovi H, Jorjani M, Ahmadiani A, Ghanavi J, Seifalian AM (2008). Properties of the amniotic membrane for potential use in tissue engineering. Eur Cell Mater.

[6] Dua HS, Azuara-Blanco A (1999). Amniotic membrane transplantation. Br J Ophthalmol.

[7] Parolini O, Alviano F, Bagnara GP (2008). Concise review: isolation and characterization of cells from human term placenta: outcome of the first international Workshop on Placenta Derived Stem Cells. Stem Cells.

[8] Allen CL, Clare G, Stewart EA (2013). Augmented dried versus cryopreserved amniotic membrane as an ocular surface dressing. PLoS One.

[9] Russo A, Bonci P, Bonci P (2012). The effects of different preservation processes on the total protein and growth factor content in a new biological product developed from human amniotic membrane. Cell Tissue Bank.

[10] Wagner M, Walter P, Salla S (2018). Cryopreservation of amniotic membrane with and without glycerol additive. Graefes Arch Clin Exp Ophthalmol.

[11] Kim TG, Do Ki K, Lee MK, So JW, Chung SK, Kang J (2019). Comparison of cytokine expression and ultrastructural alterations in fresh-frozen and dried electron beam-irradiated human amniotic membrane and chorion. Cell Tissue Bank.

[12] Paolin A, Trojan D, Leonardi A, Mellone S, Volpe A, Orlandi A, Cogliati E (2016). Cytokine expression and ultrastructural alterations in fresh-frozen, freeze-dried and γ-irradiated human amniotic membranes. Cell Tissue Bank.

[13] Maral T, Borman H, Arslan H, Demirhan B, Akinbingol G, Haberal M (1999). Effectiveness of human amnion preserved long-term in glycerol as a temporary biological dressing. Burns.

[14] Keelan JA, Sato T, Mitchell MD (1997). Interleukin (IL)-6 and IL-8 production by human amnion: regulation by cytokines, growth factors, glucocorticoids, phorbol esters, and bacterial lipopolysaccharide. Biol Reprod.

